# Supervised and extended restart in random walks for ranking and link prediction in networks

**DOI:** 10.1371/journal.pone.0213857

**Published:** 2019-03-20

**Authors:** Woojeong Jin, Jinhong Jung, U. Kang

**Affiliations:** 1 University of Southern California, Los Angeles, California, United States of America; 2 Seoul National University, Seoul, Korea; Mathematical Institute, HUNGARY

## Abstract

Given a real-world graph, how can we measure relevance scores for ranking and link prediction? Random walk with restart (RWR) provides an excellent measure for this and has been applied to various applications such as friend recommendation, community detection, anomaly detection, etc. However, RWR suffers from two problems: 1) using the same restart probability for all the nodes limits the expressiveness of random walk, and 2) the restart probability needs to be manually chosen for each application without theoretical justification. We have two main contributions in this paper. First, we propose Random Walk with Extended Restart (RWER), a random walk based measure which improves the expressiveness of random walks by using a distinct restart probability for each node. The improved expressiveness leads to superior accuracy for ranking and link prediction. Second, we propose SuRe (Supervised Restart for RWER), an algorithm for learning the restart probabilities of RWER from a given graph. SuRe eliminates the need to heuristically and manually select the restart parameter for RWER. Extensive experiments show that our proposed method provides the best performance for ranking and link prediction tasks.

## 1 Introduction

How can we measure effective node-to-node proximities for graph mining applications such as ranking and link prediction? Measuring relevance (i.e., proximity or similarity) scores between nodes is a fundamental tool for many graph mining applications [1, 2, 3, 4, 5]. Among various relevance measures, Random Walk with Restart (RWR) [6] provides useful node-to-node relevance scores by considering global network structure [7] and intricate edge relationships [8]. RWR has been successfully exploited in a wide range of data mining applications such as ranking [9, 10, 11], link prediction [1, 2, 3, 12, 5], community detection [13, 14], anomaly detection [15], etc.

However, RWR has two challenges for providing more effective relevance scores. First, RWR assumes a fixed restart probability on all nodes, i.e., a random surfer jumps back to the query node with the same probability regardless of where the surfer is located. This assumption prevents the surfer from considering the query node’s preferences for other nodes, thereby limiting the expressiveness of random walk for measuring good relevance scores. Second, RWR requires users to heuristically select the restart probability parameter without strong theoretical justification to choose the parameter.

In this paper, we propose a novel relevance measure Random Walk with Extended Restart (RWER), an extended version of RWR, which reflects a query node’s preferences on relevance scores by allowing a distinct restart probability for each node. We also propose a supervised learning method SuRe (Supervised Restart for RWER) that automatically finds optimal restart probabilities in RWER from a given graph. Extensive experiments show that our method provides the best link prediction accuracy. Table 1 summarizes strength of SuRe compared to existing methods. Our main contributions are summarized as follows:

- **Model**. We propose Random Walk with Extended Restart (RWER), a new random walk model to improve the expressiveness of RWR. RWER allows each node to have a distinct restart probability so that the random surfer has a finer control on preferences for each node.
- **Learning**. We propose SuRe, an algorithm for learning the restart probabilities in RWER from data. SuRe automatically determines the best restart probabilities.
- **Experiment**. We empirically demonstrate that our proposed method improves accuracy in all dataset.

**Table 1:** Comparison of our proposed SuRe and existing methods with respect to various aspects in ranking and link prediction tasks. SuRe outperforms all learning methods in terms of accuracy, speed, scalability, and memory usage.

Method	Supervised?	Accuracy	Speed	Scalability	Memory Usage
QUINT [12]	Yes	High	Low	Low	High
SRW [5]	Yes	High	Low	Low	Low
RWR [6]	No	Low	High	High	Low
**SuRe**	**Yes**	**Higher**	**High**	**High**	**Low**

The source code of our method and datasets used in this paper are available at https://github.com/datalabsnu/sure. The rest of this paper is organized as follows. Section 2 presents a preliminary on RWR and defines the problem. Our proposed methods are described in Section 3. After presenting experimental results in Section 4, we provide a review on related works in Section 5. Lastly, we conclude in Section 6.

## 2 Preliminaries

In this section, we describe the preliminaries on Random Walk with Restart. Then, we formally define the problem handled in this paper. We use *A*_*ij*_ or *A*(*i*, *j*) to denote the entry at the intersection of the *i*-th row and *j*-th column of matrix **A**. The *i*-th element of the vector **x** is denoted by *x*_*i*_. Table 2 lists the symbols used in this paper.

**Table 2:** Table of symbols.

Symbol	Definition
G	input graph
*n*	number of nodes in G
*m*	number of edges in G
*s*	query node (= seed node)
*c*	restart probability
**c**	(*n* × 1) restart probability vector
**r**	(*n* × 1) relevance vector
**o**	(*n* × 1) origin vector
**A**	(*n* × *n*) adjacency matrix of G
$\tilde{A}$	(*n* × *n*) row-normalized matrix of G
**A**(*i*,:)	(1 × *n*) *i*-th row of a matrix **A**
**A**(:, *j*)	(*n* × 1) *j*-th column of a matrix **A**
**J**^*ij*^	(*n* × *n*) single-entry matrix whose (*i*, *j*) entry is 1
**q**	(*n* × 1) starting vector
*P*, *N*	set of positive and negative nodes
∘	Hadamard product

### 2.1 Random walk with restart

Random walk with restart (also known as Personalized PageRank, PPR with a single seed node) [9] measures each node’s proximity (relevance) w.r.t. a given query node *s* in a graph. RWR assumes a random surfer who starts at node *s*. The surfer moves to one of its neighboring nodes with probability 1 − *c* or restarts at node *s* with probability *c*. When the surfer moves from *u* to one of its neighbors, each neighbor *v* is selected with a probability proportional to the weight in the edge (*u*, *v*). The relevance score between seed node *s* and node *u* is the stationary probability that the surfer is at node *u*. If the score is large, we consider that nodes *s* and *u* are highly related.

#### Limitations

**Fig 1:**
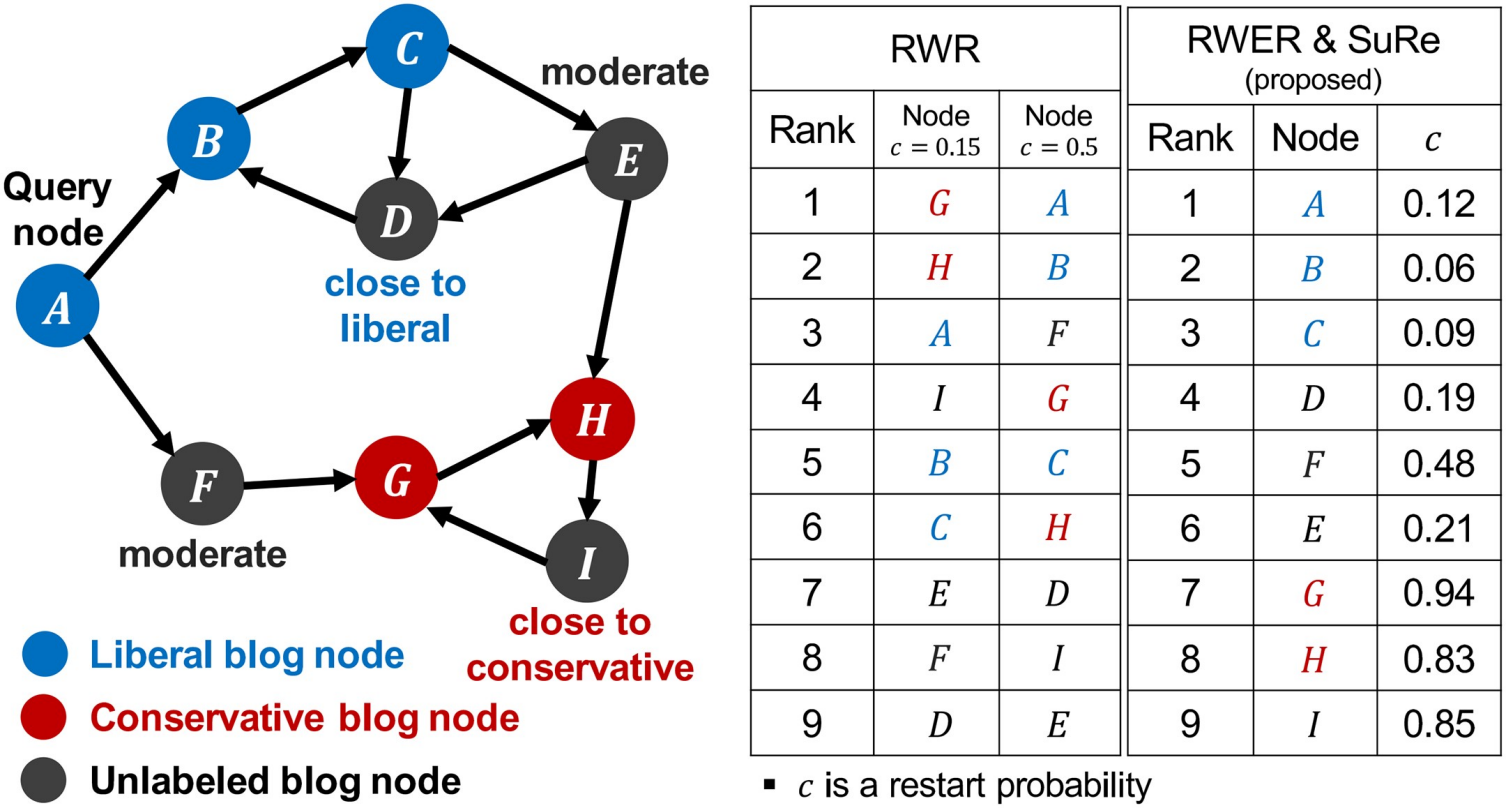
Example of RWR and our proposed approaches RWER & SuRe on a political blog network. RWR uses the fixed restart probability **0.15** or **0.5** while our proposed RWER uses distinct restart probabilities on nodes.

RWR cannot consider a query node’s preferences for estimating relevance scores between the query node and other nodes. For example, suppose we compute relevance scores from the query node *A* to other nodes in a political blog network in Fig 1 where blue colored nodes (A, B, and C) are liberal blogs, red colored ones (G and H) are conservative, black ones (D, E, F, and I) are not labeled, and an edge between nodes indicates a hyperlink between the corresponding blogs. Based on the topology of the graph, we consider that nodes *E* and *F* tend to be moderate, node *D* is likely to be liberal, and node *I* is likely to be conservative. Since the query node *A* is a liberal blog, node *A* will prefer other liberal nodes to conservative nodes. However, a conservative node *G* is ranked higher than nodes related to liberal blogs such as nodes *C* and *D* in the ranking result of RWR as shown in the left table of Fig 1. The reason is that preferences are not considered in RWR, and the random surfer jumps back to the query node *A* with a fixed restart probability *c* wherever the surfer is. On the other hand, RWER reflects the query node’s preferences on relevance scores by allowing a distinct restart probability for each node as shown in the right table of Fig 1.

Another practical problem is that it is non-trivial to set an appropriate value of the restart probability *c* for different applications since we need to manually choose *c* so that the restart probability provides optimal relevance scores for each application. RWR scores are highly affected by the restart probability; the ranking results of each restart probability (*c* = 0.15 and *c* = 0.5) are quite different as seen in the left table of Fig 1. In contrast, our learning method SuRe automatically determines the optimal restart probabilities for all nodes based on the query node’s preferences as well as relationships between nodes. The ranking result by our proposed RWER and SuRe is more desirable for the query node *A* (liberal) than those of RWR because many liberal blog nodes are ranked high in the ranking result as shown in the right table of Fig 1. The detailed descriptions of our proposed approaches RWER and SuRe are presented in Section 3.

### 2.2 Problem definition

We are given a graph G with *n* nodes and *m* edges, a query node *s*, and side information from the query node. The side information contains a set of *positive* nodes *P* = {*x*_1_, …, *x*_*k*_} that *s* prefers, and a set of *negative* nodes *N* = {*y*_1_, …, *y*_*l*_} that *s* dislikes. Our task is to learn restart probabilities for all nodes such that relevance scores of the positive nodes are greater than those of the negative ones.

## 3 Proposed method

In this section, we first describe Random Walk with Extended Restart (RWER), our proposed model for extended restart probabilities. We then propose SuRe, an efficient algorithm for learning the restart probabilities.

### 3.1 Overview of Random Walk with Extended Restart

RWER is a novel relevance model reflecting a query node’s preferences on relevance scores. The main idea of RWER is that we introduce a restart probability *vector* each of whose entry corresponds to a restart probability at a node, so that the restart probabilities are related to the preferences for the nodes.

In RWER, a restart probability of each node is interpreted as the degree of boredom of a node w.r.t. the query node. That is, if the restart probability on a node is large, then the surfer runs away from the current node to the query node (i.e., the surfer becomes bored at the node). On the other hand, if the restart probability of the node is small, then surfer desires to move around the node’s neighbors (i.e., the surfer has an interest in the node and its neighbors).

As depicted in Fig 1, each node has its own restart probability in our model RWER. The restart probabilities are determined by our supervised learning method SuRe (Section 3.5) from the query (liberal) node *A*, the positive (liberal) nodes *B* and *C*, and the negative (conservative) nodes *G* and *H*. Note that a ranking list where many liberal nodes are ranked high is desirable for the query node *A* since *A* is liberal. As shown in Fig 1, using distinct restart probabilities for each node by RWER provides more satisfactory rankings for the query node than using a single restart probability for all nodes by RWR. The restart probabilities of liberal nodes are smaller than those of conservative nodes, which implies that the random surfer prefers searching around the liberal nodes such as *B* and *C* while the surfer is likely to run away from the conservative nodes such as *G* and *H*.

One might think that it is enough to simply assign small restart probabilities to positive nodes and large restart probabilities to negative nodes for a desirable ranking. However, the restart probabilities should be determined also for unlabeled nodes, and the probabilities should reflect intricate relationships between nodes as well as the query node’s preferences. For example, the restart probability of node *F* in Fig 1 is relatively moderate because node *F* is located between a liberal node *A* and a conservative node *G*. Also, the restart probability of node *D* is small since node *D* is close to other liberal nodes *B* and *C*. Similarly, the restart probability of node *I* is large since node *I* is closely related to other conservative nodes *G* and *H*.

### 3.2 Formulation of Random Walk with Extended Restart

**Fig 2:**
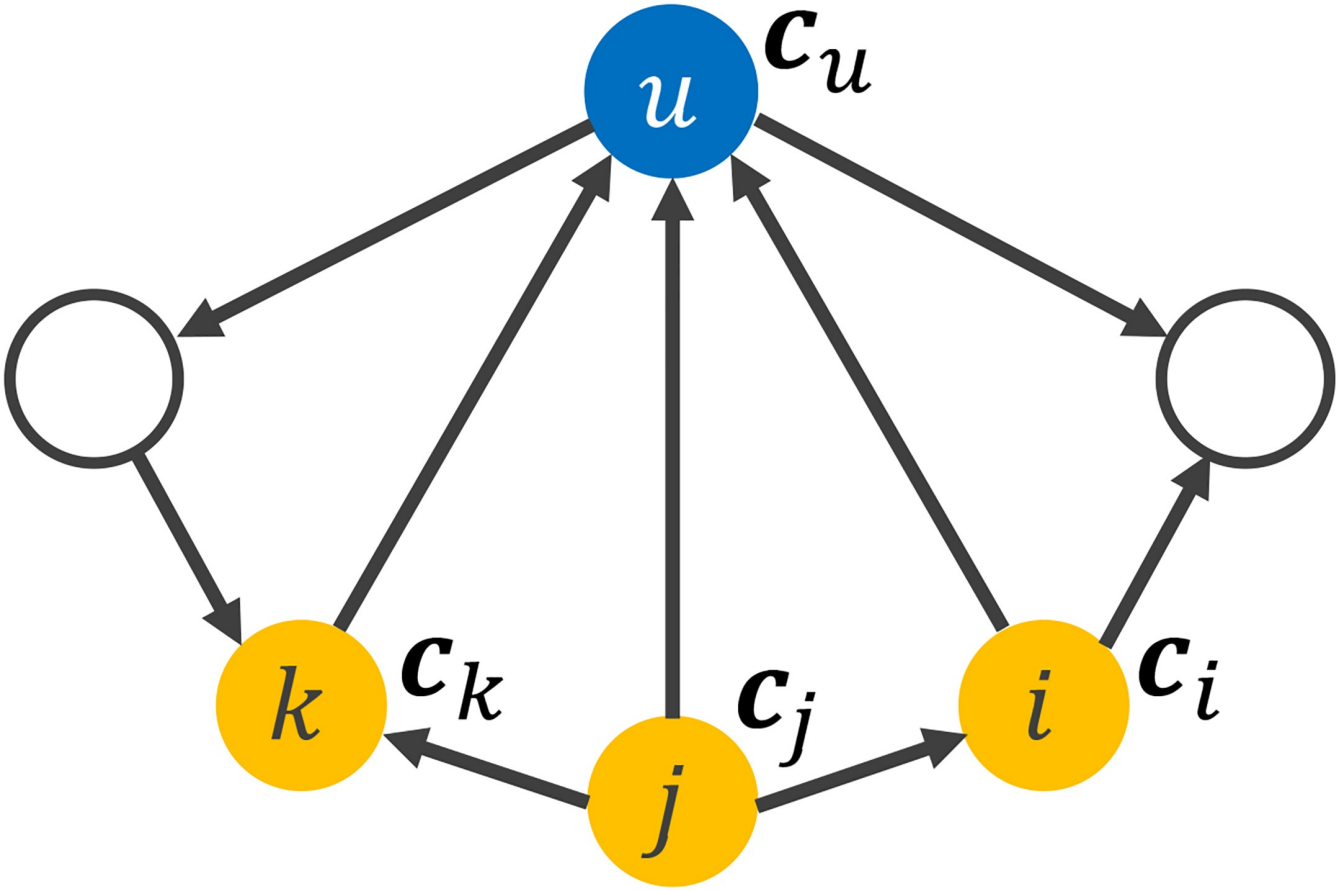
Example of a network. Each node has its own restart probability.

We formulate RWER in this section. We first explain the formulation using the example shown in Fig 2, and present general equations. In the example, the surfer goes to one of its neighbors or jumps back to the query node. To obtain the RWER probability *r*_*u*_ at time *t* + 1, we should take into account the scores of the three nodes which are *i*, *j* and *k* at time *t*. Suppose the surfer is at the node *i* at time *t*. The surfer can go to an out-neighbor through one of the two outgoing edges with probability 1 − *c*_*i*_. Note that every node has a distinct restart probability and node *i* has a restart probability *c*_*i*_ in this case. Without the restart action, $r_{u}^{\left(t+1\right)}$ in Fig 2 is defined as follows:

$$\begin{array}{c} r_{u}^{\left(t+1\right)}\leftarrow \left(1-c_{i}\right)\frac{r_{i}^{\left(t\right)}}{2}+\left(1-c_{j}\right)\frac{r_{j}^{\left(t\right)}}{3}+\left(1-c_{k}\right)r_{k}^{\left(t\right)} \end{array}$$

Also, the surfer on any node *v* jumps back to the query node with probability *c*_*v*_. The above equation is rewritten as follows considering the restart action of the random surfer:

$$\begin{array}{c} \begin{array}{cc} r_{u}^{\left(t+1\right)} & \leftarrow \left(1-c_{i}\right)\frac{r_{i}^{\left(t\right)}}{2}+\left(1-c_{j}\right)\frac{r_{j}^{\left(t\right)}}{3}+\left(1-c_{k}\right)r_{k}^{\left(t\right)}\\ & +(c_{1}r_{1}^{\left(t\right)}+\cdots +c_{v}r_{v}^{\left(t\right)}+\cdots +c_{n}r_{n}^{\left(t\right)})1\left(u=s\right) \end{array} \end{array}$$

where 1(*u* = *s*) is 1 if *u* is the query node *s*; otherwise, it is 0. Note that the restart term is different from that of the traditional random walk with restart.

Based on the aforementioned example, the recursive equation of our model is defined as follows:

$$\begin{array}{c} r_{u}=(\sum_{v\in {\text{IN}}_{u}}\left(1-c_{v}\right)\frac{r_{v}}{|{\text{OUT}}_{v}|})+(\sum_{v}c_{v}r_{v})1\left(u=s\right) \end{array}$$ (1)

where **IN**_*i*_ is the set of in-neighbors of node *i*, and **OUT**_*i*_ is the set of out-neighbors of node *i*.

Eq (1) is expressed in the form of a matrix equation as follows:

$$\begin{array}{c} r={\tilde{A}}^{⊤}\left(I-diag\left(c\right)\right)r+(c^{⊤}r)q \end{array}$$ (2)

where $\tilde{A}$ is a row-normalized matrix of the adjacency matrix **A**, **c** is a restart vector whose *i*-th entry is *c*_*i*_, *diag*(**c**) is a matrix whose *diag*(**c**)_*ii*_ = *c*_*i*_ and other entries are 0, and **q** is a vector whose *s*-th element is 1 and all other elements are 0. The random surfer can return to a set of nodes *S* = {*s*_1_, *s*_2_, …, *s*_*m*_}. In this case, *q*(*s*_*i*_) = 1/|*S*|, for *i* = 1, …, *m*. Notice that if **c** is a vector all of whose elements are the same, then the RWER is equal to RWR (or PPR). The following lemma shows that Eq (2) can be represented as a closed form equation.

**Lemma 1** *The closed form w.r.t*. **r** *in* Eq (2) *is represented as follows*:

$$\begin{array}{c} r={\left(I-B\right)}^{-1}q \end{array}$$ (3)

*where* **B** *is* ${\tilde{A}}^{⊤}\left(I-diag\left(c\right)\right)+q{\left(c-1\right)}^{⊤}$, $\tilde{A}$ *is a row-normalized matrix, and* **1** *is an all-ones vector*.

***Proof 1*** *Note that* **c**^⊤^**r** *is a scalar; thus*, (**c**^⊤^**r**)**q** = **q**(**c**^⊤^**r**) = (**qc**^⊤^)**r**. *Hence*, Eq (2) *is represented as follows*:

$$\begin{array}{cc} r & ={\tilde{A}}^{⊤}\left(I-diag\left(c\right)\right)r+(qc^{⊤})r\\ & ={\tilde{A}}^{⊤}\left(I-diag\left(c\right)\right)r+(qc^{⊤})r-q+q \end{array}$$

*Since* **r** *is a probability vector and* **1**^⊤^**r** = 1, *the above equation is written in the following equation*:

$$\begin{array}{cc} r & ={\tilde{A}}^{⊤}\left(I-diag\left(c\right)\right)r+(qc^{⊤})r-q(1^{⊤}r)+q\\ & ={\tilde{A}}^{⊤}\left(I-diag\left(c\right)\right)r+(q{\left(c-1\right)}^{⊤})r+q\\ & =({\tilde{A}}^{⊤}\left(I-diag\left(c\right)\right)+q{\left(c-1\right)}^{⊤})r+q\\ & =Br+q \end{array}$$

*where* **B** *is* ${\tilde{A}}^{⊤}\left(I-diag\left(c\right)\right)+q{\left(c-1\right)}^{⊤}$. *Finally*, **r** *is represented in the following closed form*:

$$\begin{array}{c} r={\left(I-B\right)}^{-1}q \end{array}$$

*Note that* **I** − **B** *is invertible (see the following Lemma 2)*.

**Lemma 2** *Suppose* **B** *is* ${\tilde{A}}^{⊤}\left(I-diag\left(c\right)\right)+q{\left(c-1\right)}^{⊤}$. *Then*, **M** = **I** − **B** *is invertible*.

***Proof 2*** *Let* **C** *be* **I** − *diag*(**c**), *and* **X** *be* $I-{\tilde{A}}^{⊤}C$. *Then*, **M** = **I** − **B** *is represented as follows*:

$$\begin{array}{cc} M & =I-{\tilde{A}}^{⊤}C-q{\left(c-1\right)}^{⊤}\\ & =X+q{\left(1-c\right)}^{⊤} \end{array}$$

*Note that* ${\left({\tilde{A}}^{⊤}C\right)}_{ij}\geq 0$, 1 ≤ *i*, *j* ≤ *n and the largest eigenvalue* $\lambda_{max}\left({\tilde{A}}^{⊤}C\right)\leq 1$ *since* $\tilde{A}$ *is stochastic and* **C** *is sub-stochastic*. *In other words*, $X=I-{\tilde{A}}^{⊤}C$ *is M-matrix* [16]; *thus*, **X**^−1^ *exists and all entries of* **X**^−1^ *are nonnegative*. *By Sherman-Morrison lemma* [17], (**X** + **q**(**1** − **c**)^⊤^)^−1^ *is represented as follows*:

$$\begin{array}{c} (X+q{\left(1-c\right)}^{⊤}{)}^{-1}=X^{-1}-\frac{X^{-1}q{\left(1-c\right)}^{⊤}X^{-1}}{1+{\left(1-c\right)}^{⊤}X^{-1}q} \end{array}$$

*Since all entries of* **X**^−1^, **1** − **c** *and* **q** *are nonnegative*, (**1** − **c**)^⊤^ **X**^−1^ **q** ≥ 0; *thus*, 1 + (**1** − **c**)^⊤^ **X**^−1^ **q** ≥ 1. *Hence, the right side of the above equation exists, i.e*., (**X** + **q**(**1** − **c**)^⊤^)^−1^ = **M**^−1^ *exists*. **I** − **B**^⊤^ *is also invertible, which is proved similarly to the proof of* **I** − **B**.

Note that if **c** is given, the RWER vector **r** can be calculated using the closed form in Lemma 1. However, the computation using the closed form requires *O*(*n*^3^) time and *O*(*n*^2^) memory space due to the matrix inversion where *n* is the number of nodes; thus, this approach is impractical when we need to compute RWER scores in large-scale graphs. In order to avoid the heavy computational cost, we exploit an efficient iterative algorithm described in Section 3.3.

### 3.3 Algorithm for Random Walk with Extended Restart

We present an iterative algorithm for computing RWER scores efficiently. Our algorithm is based on power iteration and comprises two phases: a normalization phase (Algorithm 1) and an iteration phase (Algorithm 2).

#### Normalization phase (Algorithm 1)

Our proposed algorithm first computes the out-degree diagonal matrix **D** of **A** (line 1). Then, the algorithm computes the row normalized matrix $\tilde{A}$ using **D** (line 2).

#### Iteration phase (Algorithm 2)

Our algorithm computes the RWER score vector **r** for the seed node *s* in the iteration phase. As described in Section 3.2, the vector **q** denotes a length-*n* starting vector whose entry at the index of the seed node is 1 and otherwise 0 (line 1). Our algorithm iteratively computes Eq (2) (line 3). We then compute the error *δ* between **r**′, the result in the previous iteration, and **r** (line 4). Next, we update **r**′ into **r** for the next iteration (line 5). The iteration stops when the error *δ* is smaller than a threshold *ϵ* (line 6).

#### Theoretical analysis

We analyze the convergence of the iterative algorithm in Theorem 1 and the time complexity in Theorem 2. We assume that all the matrices considered are saved in a sparse format, such as the compressed column storage [18], which stores only non-zero entries, and that all the matrix operations exploit such sparsity by only considering non-zero entries.

**Algorithm 1** Normalization phase of RWER

**Input**: adjacency matrix **A**

**Output**: row-normalized matrix $\tilde{A}$

1: compute a degree diagonal matrix **D** of **A** (i.e., **D**_*ii*_ = ∑_*j*_ **A**_*ij*_)

2: compute a normalized matrix, $\tilde{A}=D^{-1}A$.

3: **return** $\tilde{A}$

**Algorithm 2** Iteration phase of RWER

**Input**: row-normalized matrix $\tilde{A}$, query node *s*, restart probability vector **c**, and error tolerance *ϵ*

**Output**: RWER score vector **r**

1: set the starting vector **q** from the seed node *s*

2: **repeat**

3:  $r^{\prime }\leftarrow {\tilde{A}}^{⊤}(I-diag(c))r+(c^{⊤}r)q$

4:  compute error, *δ* = ‖**r**′ − **r**‖

5:  update **r** ← **r**′

6: **until** *δ* < *ϵ*

7: **return r**

**Theorem 1 (Convergence)** *Suppose the graph represented by* $\tilde{A}$ *is irreducible and aperiodic. Then, the power iteration algorithm (Algorithm 2) for RWER converges*.

***Proof 3*** Eq (2) *is represented as follows*:

$$\begin{array}{c} \begin{array}{cc} r & ={\tilde{A}}^{⊤}\left(I-diag\left(c\right)\right)r+(qc^{⊤})r\\ & =({\tilde{A}}^{⊤}\left(I-diag\left(c\right)\right)+(qc^{⊤}))r=Gr \end{array} \end{array}$$

*Note that* **G** *is a column stochastic matrix*. *Moreover*, **G** *is irreducible since* $\tilde{A}$ *is irreducible, and aperiodic due to the self-loop at node s by restart*. *Hence*, **r** *is the eigenvector corresponding to the principal eigenvalue of* **G**, *and the power iteration for* **r** *converges* [19].

**Theorem 2 (Time Complexity)** *The time complexity of Algorithm 2 is O*(*Tm*) *where T is the number of iterations, and m is the number of edges*.

***Proof 4*** *We assume that the number of edges is greater than that of nodes for simplicity. The iterative algorithm for computing RWER scores takes O*(*Tm*) *time since each iteration requires the sparse matrix-vector multiplication which takes O*(*m*) *time*.

Theorem 2 indicates that our method in Algorithm 2 presents the linear scalability w.r.t the number of edges.

### 3.4 Cost function

Although our relevance measure RWER improves the expressiveness of RWR by introducing a distinct restart probability for each node, it is difficult to manually investigate the optimal restart probabilities for all nodes in large graphs. In this section, we define the cost function for finding the optimal restart probabilities.

As mentioned in Section 2.2, our goal is to set the optimal restart probabilities so that the relevance scores of positive nodes outweigh those of negative nodes. We define the following cost function:

$$\begin{array}{c} \underset{c}{\text{arg}\;\text{min}}\;F\left(c\right)=\lambda {\left\|c-o\right\|}^{2}+\sum_{x\in P,y\in N}h\left(r_{y}-r_{x}\right) \end{array}$$ (4)

where λ is a regularization parameter that controls the importance of the regularization term, **o** is a given origin vector, *h* is a loss function, and *r*_*x*_ and *r*_*y*_ are RWER scores of nodes *x* and *y*, respectively. The cost function is obtained from the pairwise differences between the RWER scores of positive and negative nodes. Given an increasing loss function *h*, *F*(**c**) is minimized as the scores of positive nodes are maximized and those of negative nodes are minimized. The origin vector **o** prevents the **c** vector becoming too small, and serves as a model regularizer which helps avoid overfitting and thus improves accuracy, as we will see in Section 4.4. We set **o** to a constant vector all of whose elements are set to a constant. We use the loss function *h*(*x*) = (1 + exp(−*x*/*b*))^−1^ since the loss function maximizes AUC of binary classification when *b* is small enough [20, 5]; in that case the loss function becomes a step function.

### 3.5 SuRe—Optimizing the cost function

Our goal is to minimize Eq (4) with respect to **c**. Note that the objective function *F*(**c**) is not convex. Thus, we exploit the gradient descent method to find the local minimum of function *F*(**c**). For the purpose, we first need to obtain the derivative of *F*(**c**) w.r.t. **c**:

$$\begin{array}{c} \begin{array}{cc} \frac{\partial F(c)}{\partial c} & =2\lambda \left(c-o\right)+\sum_{x\in P,y\in N}\frac{\partial h(r_{y}-r_{x})}{\partial c}\\ & =2\lambda \left(c-o\right)+\sum_{x\in P,y\in N}\frac{\partial h(\delta_{yx})}{\partial \delta_{yx}}\left(\frac{\partial r_{y}}{\partial c}-\frac{\partial r_{x}}{\partial c}\right) \end{array} \end{array}$$ (5)

where *δ*_*yx*_ is *r*_*y*_ − *r*_*x*_. The derivative $\frac{\partial h(\delta_{yx})}{\partial \delta_{yx}}$ of the loss function is $\frac{1}{b}h\left(\delta_{yx}\right)\left(1-h\left(\delta_{yx}\right)\right)$.

In order to obtain the derivative $\frac{\partial r_{x}}{\partial c}$, we have to calculate the derivative of the relevance score *r*_*x*_ w.r.t. *c*_*i*_ which is the *i*-th element of **c**. Let **M** be (**I** − **B**)^−1^; then, **r** = (**I** − **B**)^−1^ **q** = **Mq**, **M**(:, *s*) = **r**, and *M*(*x*, *s*) = *r*_*x*_, from Theorem 1.

Since **M** is the inverse of **I** − **B**, according to [21], $\frac{\partial M}{\partial c_{i}}$ becomes:

$$\begin{array}{c} \frac{\partial M}{\partial c_{i}}=-M\frac{\partial (I-B)}{\partial c_{i}}M=M\left(-{\tilde{A}}^{⊤}J^{ii}+J^{si}\right)M \end{array}$$

where **J**^*ij*^ is a single-entry matrix whose (*i*, *j*)th entry is 1 and all other elements are 0. Based on the above equation, $\frac{\partial M(x,s)}{\partial c_{i}}$ is represented as follows:

$$\begin{array}{c} \frac{\partial M(x,s)}{\partial c_{i}}=M\left(x,:\right)\left(-{\tilde{A}}^{⊤}\left(:,i\right)+e_{s}\right)M\left(i,s\right) \end{array}$$

where **e**_*s*_ is a length *n* unit vector whose *s*-th entry is 1. Note that $\frac{\partial M(x,s)}{\partial c_{i}}$ is calculated for 1 ≤ *i* ≤ *n*; then, $\frac{\partial r_{x}}{\partial c}$ is written in the following equation:

$$\begin{array}{c} \frac{\partial r_{x}}{\partial c}=\frac{\partial M(x,s)}{\partial c}=(\left(-\tilde{A}+1e_{s}^{⊤}\right)M{\left(x,:\right)}^{⊤})\circ M\left(:,s\right) \end{array}$$ (6)

where ∘ denotes Hadamard product, and **1** is an all-ones vector of length *n*. Similarly, $\frac{\partial r_{y}}{\partial c}$ is calculated by switching *x* to *y*. Using the Eq (6), $\sum_{x\in P,y\in N}\frac{\partial h(\delta_{yx})}{\partial \delta_{yx}}\left(\frac{\partial r_{y}}{\partial c}-\frac{\partial r_{x}}{\partial c}\right)$ in Eq (5) is represented as follows:

$$\begin{array}{c} \begin{array}{cc} \left(\left(-\tilde{A}+1e_{s}^{⊤}\right)\;\;\;\;\sum_{x\in P,y\in N}\;\;\;\frac{\partial h(\delta_{yx})}{\partial \delta_{yx}}(M(y,:)-M(x,:){)}^{⊤}\right) & \circ M(:,s)\\ =(\left(-\tilde{A}+1e_{s}^{⊤}\right)\tilde{r}) & \circ r \end{array} \end{array}$$ (7)

where **r** = **M**(:, *s*) is an RWER score vector, and $\tilde{r}=\sum_{x\in P,y\in N}\;\;\frac{\partial h(\delta_{yx})}{\partial \delta_{yx}}(M(y,:)-M(x,:){)}^{⊤}$. Then, $\frac{\partial F(c)}{\partial c}$ in Eq (5) is represented as follows:

$$\begin{array}{c} \frac{\partial F(c)}{\partial c}=2\lambda \left(c-o\right)+(\left(-\tilde{A}+1e_{s}^{⊤}\right)\tilde{r})\circ r \end{array}$$ (8)

**Algorithm 3** SuRe—Learning a restart vector **c**

**Input**: adjacency matrix **A**, query node *s*, positive set *P*, negative set *N*, origin vector **o**, parameter *b* of loss function *h*, and the learning rate *η*

**Output**: the learned restart vector **c**

1: randomly initialize **c**

2: **while c** does not converge **do**

3:  compute **r** = **M**(:, *s*) based on Eq (2) (Algorithm 2)

4:  compute $\tilde{r}=\sum_{x\in P,y\in N}\frac{\partial h(\delta_{yx})}{\partial \delta_{yx}}(M(y,:)-M(x,:){)}^{⊤}$ by a linear system solver (Lemma 3)

5:  compute $\frac{\partial F(c)}{\partial c}$ by Eq (8)

6:  update $c\leftarrow c-\eta \frac{\partial F(c)}{\partial c}$

7: **end while**

8: **return** the learned restart vector **c**

Notice that we do not obtain **M** explicitly to compute **M**(:, *s*) in Eq (8) since **M** is the inverse of **I** **−** **B** and inverting a large matrix is infeasible as mentioned in Section 3.2. Instead, we use the iterative method described in Algorithm 2 to get **r** = **M**(:, *s*). However, the problem is that we also require rows of **M** (i.e., **M**(*x*,:) in $\tilde{r}$), and Algorithm 2 only computes a column of **M** for a given seed node. How can we calculate $\tilde{r}$ without inverting **M**? $\tilde{r}$ is computed iteratively by the following lemma:

**Lemma 3** *From the result of* Eq (7), $\tilde{r}=\sum_{x\in P,y\in N}\frac{\partial h(\delta_{yx})}{\partial \delta_{yx}}(M(y,:)-M(x,:){)}^{⊤}$ *which is represented as* $\tilde{r}=M^{⊤}\tilde{p}\Leftrightarrow \left(I-B^{⊤}\right)\tilde{r}=\tilde{p}$ *where* $\tilde{p}=\sum_{x\in P,y\in N}\;\;\frac{\partial h(\delta_{yx})}{\partial \delta_{yx}}\left(e_{y}-e_{x}\right)$, **e**_*x*_ *is an n* × 1 *vector whose x-th element is* 1 *and the others are* 0, *and δ*_*yx*_ = *r*_*y*_ − *r*_*x*_. *Then*, $\tilde{r}$ *is the solution of the linear system* $\left(I-B^{⊤}\right)\tilde{r}=\tilde{p}$ *which is solved by an iterative method for linear systems*.

***Proof 5*** **M**(*x*,:)^⊤^ *is a column vector which is the transpose of the x-th row of the matrix* **M**. *In other words*, $M\left(x,:\right)=e_{x}^{⊤}M\;\Leftrightarrow \;M{\left(x,:\right)}^{⊤}\;\;\;=\;M^{⊤}e_{x}$ *where* **e**_*x*_ *is an n* × 1 *vector whose x-th element is* 1 *and the others are* 0. *Then*, $\tilde{r}$ *is represented as follows*:

$$\begin{array}{cc} \tilde{r} & =\sum_{x\in P,y\in N}\frac{\partial h(\delta_{yx})}{\partial \delta_{yx}}(M(y,:)-M(x,:){)}^{⊤}\\ & =\sum_{x\in P,y\in N}\frac{\partial h(\delta_{yx})}{\partial \delta_{yx}}(M^{⊤}e_{y}-M^{⊤}e_{x})\\ & =M^{⊤}\;\;\;\;\sum_{x\in P,y\in N}\frac{\partial h(\delta_{yx})}{\partial \delta_{yx}}(e_{y}-e_{x})=M^{⊤}\tilde{p} \end{array}$$

*where* $\tilde{p}=\sum_{x\in P,y\in N}\;\;\frac{\partial h(\delta_{yx})}{\partial \delta_{yx}}\left(e_{y}-e_{x}\right)$. *Also*, $\tilde{r}$ *is represented as the following linear system*:

$$\begin{array}{c} \tilde{r}=M^{⊤}\tilde{p}={\left(I-B\right)}^{-⊤}\tilde{p}\Leftrightarrow \left(I-B^{⊤}\right)\tilde{r}=\tilde{p} \end{array}$$

*where* $B={\tilde{A}}^{⊤}\left(I-diag\left(c\right)\right)+q{\left(c-1\right)}^{⊤}$.

Note that **M**^−⊤^ = **I** − **B**^⊤^ is non-symmetric and invertible (Lemma 2); thus, any iterative method for a non-symmetric matrix can be used to solve for $\tilde{r}$. We use GMRES [22], an iterative method for solving linear systems since it is the state-of-the-art method in terms of efficiency and accuracy.

#### Optimization phase (Algorithm 3)

**Fig 3:**
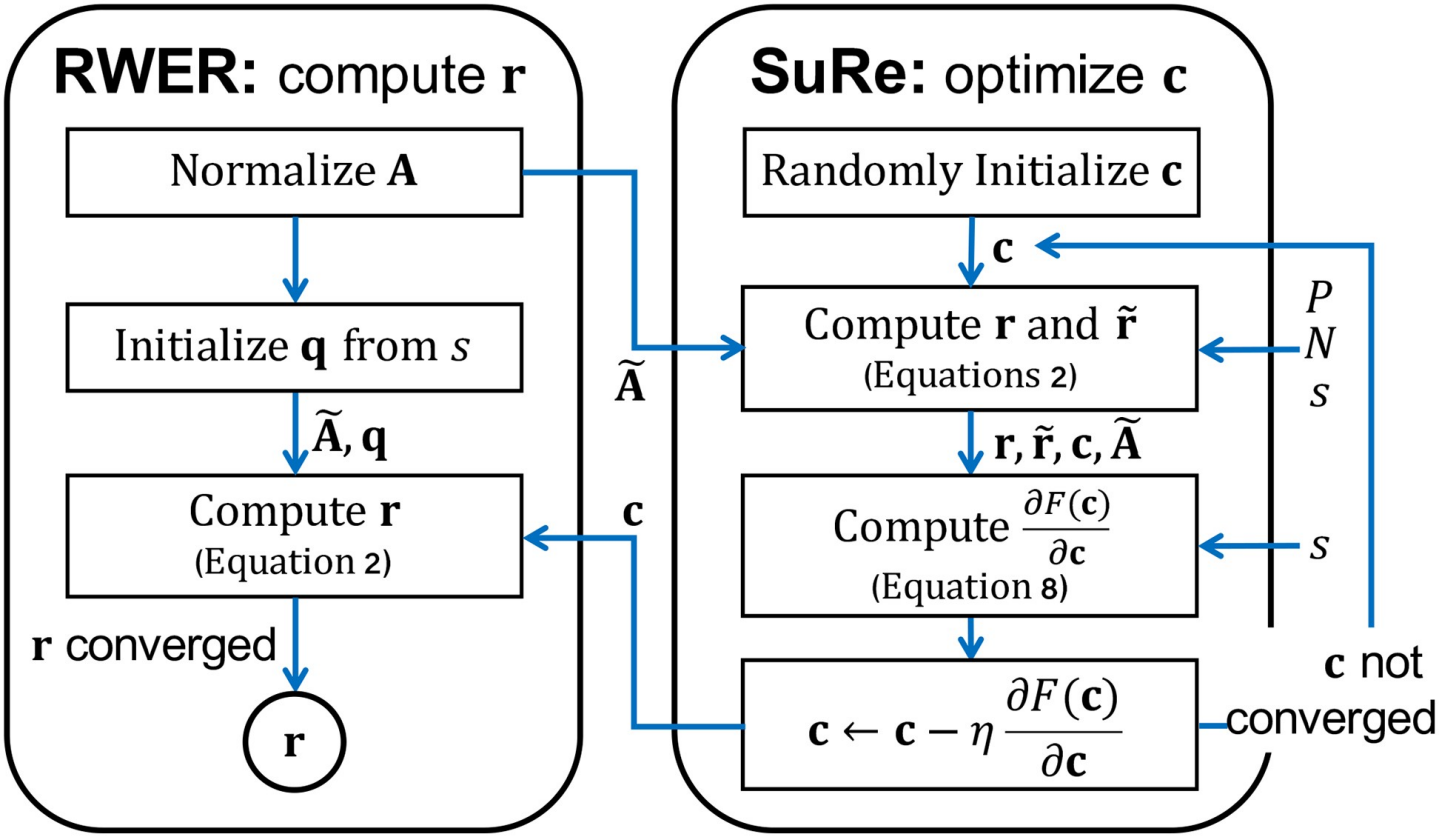
Flowchart of RWER (Algorithms 1 and 2) and SuRe (Algorithm 3). SuRe learns restart probability vector **c**, and RWER computes our node relevance score vector **r** for a given seed node *s*.

SuRe algorithm for solving the optimization problem is summarized in Algorithm 3 and Fig 3. In the algorithm, we use a gradient-based method to update the restart probability **c** based on Eq (8).

### 3.6 Theoretical analysis

We analyze the time complexity of SuRe (Algorithm 3).

**Lemma 4** *Let* |*P*| *and* |*N*| *denote the number of positive and negative nodes, respectively*. *The computation of* $\tilde{r}=\sum_{x,y}\frac{\partial h(\delta_{yx})}{\partial \delta_{yx}}{\left(M\left(y,:\right)-M\left(x,:\right)\right)}^{⊤}$ *takes O*(*Tm* + |*P*||*N*|) *time where T is the number of iterations until convergence, and m is the number of edges*.

***Proof 6*** *Note that solving a sparse linear system* **Ax** = **b** *with an iterative method such as GMRES* [22] *requires O*(*T*|**A**|) *time where T is the number of iterations, and* |**A**| *is the number of non-zeros of* **A**. *Hence, it takes O*(*T*|**I** − **B**^⊤^|) = *O*(*Tm*) *time to solve the linear system* $\left(I-B^{⊤}\right)\tilde{r}=\tilde{p}$ *by the iterative method where the number of non-zeros of* **I** − **B**^⊤^ *is bounded by O*(*m*). *In addition, setting* $\tilde{p}$ *takes O*(|*P*||*N*|) *time*. *Thus, the overall time complexity for computing* $\sum_{x,y}\frac{\partial h(\delta_{yx})}{\partial \delta_{yx}}{\left(M\left(y,:\right)-M\left(x,:\right)\right)}^{⊤}$ *is O*(*Tm* + |*P*||*N*|).

Based on Lemma 4, the time complexity of Algorithm 3 is presented in Theorem 3.

**Theorem 3 (Time complexity of Algorithm 3)** *For a given graph with m non-zero elements, the learning algorithm* SuRe *takes O*(*T*_1_(*T*_2_ *m* + |*P*||*N*|)) *time where T*_1_ *is the number of times* **c** *is updated with the gradient, and T*_2_ *is the number of inner iterations for computing* **r** *and* $\tilde{r}$.

***Proof 7*** *The computation of* **r** *takes O*(*T*′*m*) *time according to Theorem 3.2*. *The computation of* $\tilde{r}=\sum_{x,y}\frac{\partial h(\delta_{yx})}{\partial \delta_{yx}}{\left(M\left(y,:\right)-M\left(x,:\right)\right)}^{⊤}$ *takes O*(*T*″*m* + |*P*||*N*|) *time according to Lemma 4*. *Besides, the computation of* $\frac{\partial F(c)}{\partial c}$ *takes additional O*(*m*) *time due to the sparse matrix-vector multiplication*. *Hence*, SuRe *takes O*(*T*_1_(*T*_2_*m* + |*P*||*N*|)) *time where T*_1_ *is the number of iterations of the gradient descent procedure in order that the restart vector converges and T*_2_ = *T*′ + *T*″.

Theorem 3 implies that our learning method SuRe in Algorithm 3 provides linear time and space scalability w.r.t. the number *m* of edges. Notice that |*P*| and |*N*| are constants much smaller than *m*.

### 3.7 Discussion on restart probabilities

The restart vector **c** can be considered as a function of attributes, $c_{i}=H\left(w_{i}^{T}a_{i}\right)$, where **a**_**i**_ and **w**_**i**_ are an attribute vector and a weight vector of node *i*, respectively. We can incorporate rich attributes into the attribute vector from a given graph. For instance, node attributes such as each node’s degree and the number of common neighbors can be utilized. *H*(*x*) can be an exponential function *exp*(*x*) or a logistic function 1/(1 + *exp*(−*x*/*d*)). In this setting, finding good attributes is a key factor to show the best performances. However, using restart probabilities, which are independent of attributes, shows better performances due to the lack of attributes in our problem setting. Further works include learning the restart probabilities as a function of attributes.

## 4 Experiment

We evaluate our proposed method with various baseline approaches. Since there is no ground-truth of node-to-node relevance scores in real-world graphs, we instead evaluate the performance of two representative applications based on relevance scores: ranking and link prediction. Based on these settings, we aim to answer the following questions:

- **Q1. Ranking performance (Section 4.2)**. Does our proposed method SuRe provide the best relevances scores for ranking compared to other methods?
- **Q2. Link prediction performance (Section 4.3)**. How effective is SuRe for link prediction tasks?
- **Q3. Parameter sensitivity (Section 4.4)**. How do parameters used in SuRe affect the accuracy of link prediction?
- **Q4. Effects of number of labeled nodes. (Section 4.5)**. How does the number of labeled nodes affect the performance of link prediction?
- **Q5. Scalability (Section 4.6)**. How well does SuRe scale up with the number of edges?

### 4.1 Experimental settings

#### Datasets

We experiment on various real-world network datasets. Datasets used in our experiments are summarized in Table 3. We use Polblogs for the ranking task (Section 4.2), HepPh and HepTh for the link prediction task (Section 4.3), and Wikipedia for the scalability experiment (Section 4.6). The Wikipedia dataset is a hyperlink network. The HepPh and HepTh datasets are collaboration networks where nodes are authors, and edges are collaboration relationships time-stamped from May 15, 1992 to August 14, 1996 and from October 1, 1993 to December 10, 1999, respectively. The Polblogs dataset is a political network made up of liberal and conservative blogs. Since only HepPh and HepTh have time information, we use them in the link prediction task. All experiments are performed on a Linux machine with Intel(R) Xeon E5-2630 v4 CPU @ 2.2GHz and 256GB memory.

**Table 3:** Dataset statistics. The query nodes are used for the ranking and the link prediction tasks.

Dataset	#Nodes	#Edges	#Queries
Wikipedia^1^	3,023,165	102,382,410	-
HepPh^1^	34,546	421,534	135
HepTh^1^	27,770	352,768	121
Polblogs^2^	1,490	19,025	115

^1^ http://konect.uni-koblenz.de

^2^ http://www-personal.umich.edu/~mejn/netdata

**Methods**. We compare our proposed method SuRe with the following methods:

- Simple RWER method (S-RWER): sets the restart probabilities such that positively labeled nodes are given 0.1 and negatively labeled nodes are given 0.7.
- Common Neighbor (CN) [23]: |*N*_*set*_(*x*) ∩ *N*_*set*_(*y*)| where *N*_*set*_ is set of neighbors.
- Adamic-Adar (AA) [1]: $\sum_{z\in N_{set}\left(x\right)\cap N_{set}\left(y\right)}\frac{1}{log|N_{set}\left(z\right)|}$.
- Jaccard’s Coefficient (JC) [24]: $\frac{N_{set}\left(x\right)\cup N_{set}\left(y\right)}{N_{set}\left(x\right)\cap N_{set}\left(y\right)}$
- Random Walk with Restart (RWR) [6]: performs RWR on the network with no side information used.
- Supervised Random Walks (SRW) [5]: learns a function that assigns weights to edges. We use each node’s degree and the number of common neighbors as features.
- QUINT [12]: learns an adjacency matrix which represents edge’s weights and presence of edges.
- Prioritization algorithm [25]: prioritizes proteins in protein-protein interaction graphs.
- SimRank [26]: measures similarity of nodes based on their relationships with other nodes.

#### Parameters

We set the origin vector **o** in SuRe and restart probability *c* in RWR, SRW, and QUINT to the ones that give the best performance. Also, in SuRe, SRW, and QUINT, we set λ = 1 among {0.01, 0.1, 1, 10}, and *b* = 10^−2^ among {10^−1^, 10^−2^, 10^−3^, 10^−4^} by grid search. The selected parameters give the best performance for each method.

#### Evaluation metrics

To compare the methods, we use Mean Average Precision (MAP), Area under the ROC curve (AUC), and Precision@20. MAP is the mean of average precisions for multiple queries. AUC is the expectation that a uniformly drawn random positive is ranked higher than a uniformly drawn random negative. Precision@20 is the precision at the top-20 position in a ranking result. The higher the values of the metrics are, the better the performance is.

### 4.2 Ranking performance

We evaluate the ranking performance of our method SuRe compared to that of other methods.

#### Experimental setup

We perform this experiments on the Polblogs dataset. In Polblogs dataset, a node represents a blog, and an edge between nodes indicates a hyperlink between blogs. In the dataset, each node has a label which is either *liberal* or *conservative*. Among nodes connected from the query node (i.e., neighbors), we choose nodes having the same political position to the query node as positive nodes, and nodes having the opposite propensity to the query node as negative nodes. Note that the numbers of positive nodes and negative nodes do not exceed their query node’s degree. We sample 115 nodes whose degrees are greater than 4 as query nodes to perform this experiment. We use all nodes except neighbors from a query node as test nodes. In this experiment, we aim to boost ranks of nodes having the same political position as the query node.

#### Case study

We analyze the ranking quality produced from each method in the Polblogs dataset. Table 4 shows the top-10 ranking list for a query node *obsidianwings*, a liberal blog. Red colored nodes are conservative, and the black colored ones are liberal. As shown in the table, our ranking result from SuRe is of a higher quality compared to those from RWR, SRW, and QUINT since top-10 ranking result from SuRe contains only liberal nodes while other ranking results have several conservative nodes, considering that the query node is liberal.

**Table 4:** Ranking results of our proposed method SuRe and other methods w.r.t. a query node *obsidianwings*, a liberal blog. Bold nodes are conservative blogs, and the non-bold ones are liberal. Our ranking result from SuRe contains only liberal nodes, indicating the best result, while other ranking results wrongly contain conservative nodes.

Rank	SuRe	RWR	SRW	QUINT	Prioriti-zation	AA	JC	CN	Sim-Rank
1	digbys	**freere**	digbys	**freere**	**freere**	**dalyth**	amptoo	**dalyth**	davids
2	billmo	**michel**	tbogg	**michel**	politi	newlef	thepoo	thepoo	religi
3	gadfly	**prolif**	libera	**prolif**	**kausfi**	thepoo	**tagord**	madkan	**asmall**
4	reach	**rightw**	billmo	**rightw**	gadfly	madkan	oddhou	aintno	liquid
5	jamesw	digbys	xnerg	digbys	**jewish**	amptoo	althip	politi	marksc
6	angryb	**little**	corren	**little**	**prolif**	politi	oxblo	amptoo	profgo
7	marks	billmo	**hughhe**	billmo	**rightw**	southk	aintno	busybu	uggabu
8	tbogg	jamesw	busybu	jamesw	jamesw	aintno	**balloo**	**tagord**	needle
9	wampu	reachm	pacifi	reachm	dailyh	busybu	**pejman**	billmo	stalini
10	steveg	politi	nielse	**hughhe**	asmall	**balloo**	strang	libera	amptoo

#### Result

To evaluate ranking performances, we measure MAP, Precision@20, and AUC for the ranking results produced from our method SuRe and other methods.

**Fig 4:**
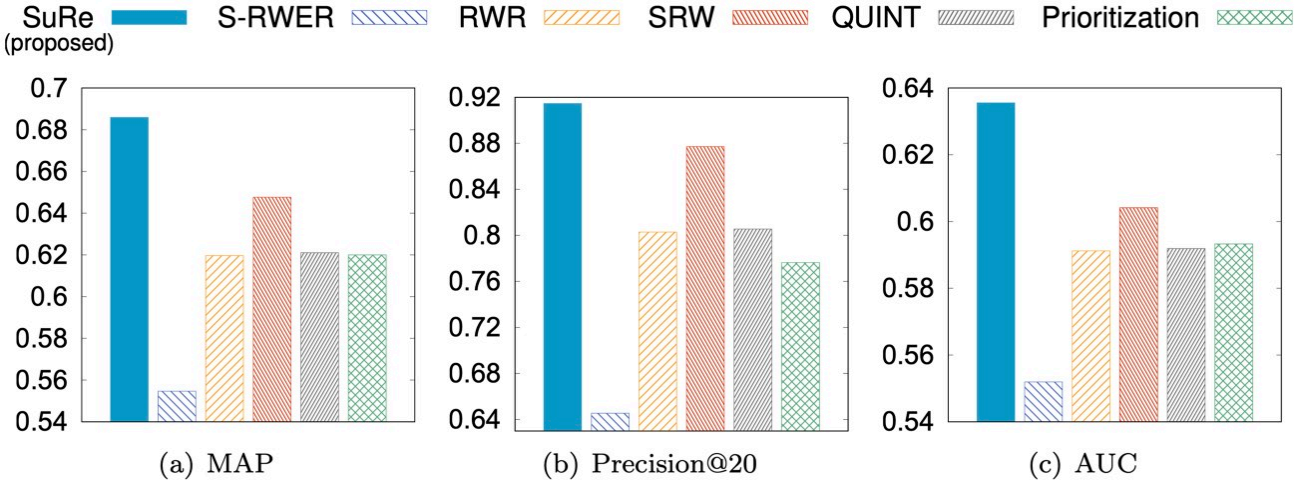
Ranking performance on Polblogs. Our method SuRe provides the best ranking performance compared to existing methods in terms of MAP and Precision@20. Note that the maximal values are 1 for each plot.

In Polblogs, if the query node is liberal (conservative), then the positive class is liberal (conservative), and the negative one is conservative (liberal). As shown in Fig 4, our method SuRe shows the best ranking performance compared to other methods in terms of MAP, Precision@20, and AUC. Also, SuRe shows better performances over the simple RWER method (S-RWER), which means SuRe learns the restart probabilities effectively.

### 4.3 Link prediction performance

We examine the link prediction performance of our proposed method SuRe compared to other link prediction methods as well as RWR-based methods SRW and QUINT.

#### Experimental setup

**Fig 5:**
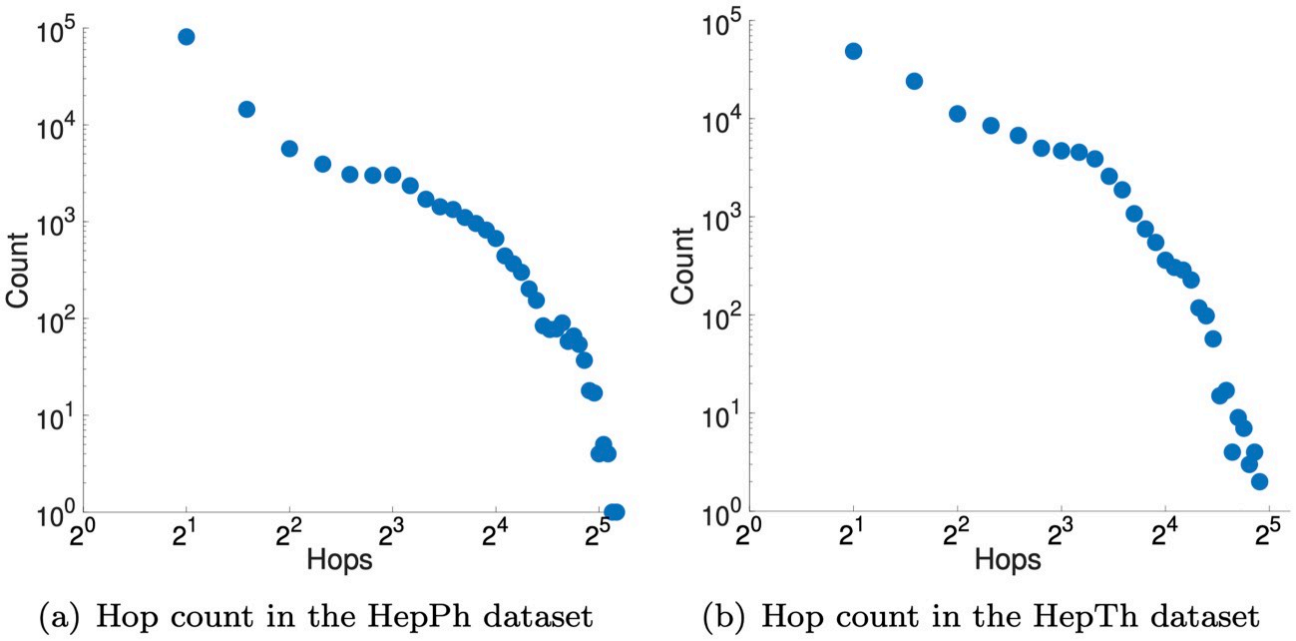
Hop count plot in the datasets used in the link prediction task. Note that the majority of nodes are within 2 hops in both real-world networks.

In the link prediction task, we aim to predict future links from a query node to other nodes based on relevance scores. We perform this experiments on the HepPh and HepTh datasets which are time-stamped networks. We follow the setting of [5] and focus on predicting links to nodes that are 2-hops away from the query node since most of new edges are created closing a triangle. Fig 5 supports the triangle closing setting used in the link prediction task. When an edge is newly added, we count the hop between two nodes to be connected by the edge. Note that the majority of the connected nodes are 2 hops away in both of the datasets.

The detailed experimental setting of the link prediction task is as follows:

- We consider time-stamp information in networks to construct training and test datasets as in [5]. For each query node *s*, let *t*_*s*,min_ and *t*_*s*,max_ denote the minimum and maximum time-stamp of 1-hop neighbors of node *s*, respectively. Suppose *t*_*s*,min_ < *t*_*s*,1_ < *t*_*s*,2_ < *t*_*s*,max_. We select links (*s*, *v*) created between *t*_*s*,2_ and *t*_*s*,max_ as test data where node *v* is 2-hop neighbors from node *s* before the links are created. We choose the query node’s 1-hop neighbors created between *t*_*s*,1_ and *t*_*s*,2_ as positive nodes. We sample the same number of negative nodes as that of positive ones, which are 3-hops or more from node *s*. We exploit other links excepts the test links as training data. We select *t*_*s*,1_ and *t*_*s*,2_ such that *t*_*s*,1_ = *t*_*s*,min_ + 0.3*L* and *t*_*s*,2_ = *t*_*s*,min_ + 0.7*L*, respectively, where *L* = *t*_*s*,max_ − *t*_*s*,min_ is the total time length.

**Fig 6:**
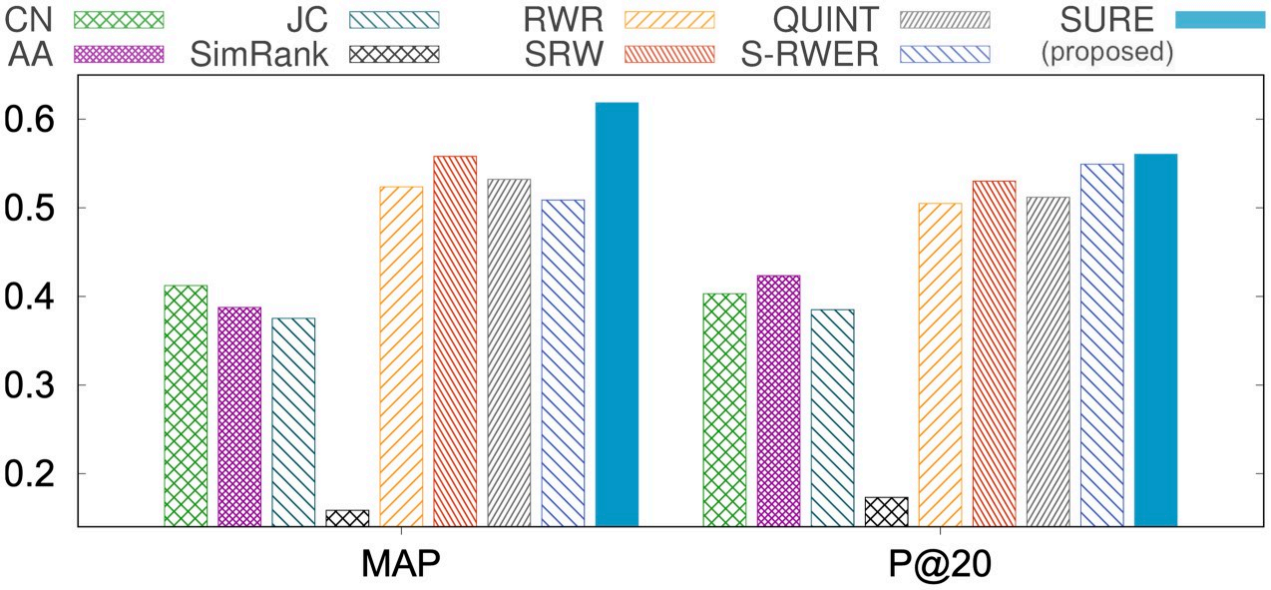
Link prediction performance on HepPh dataset, where the degree of each query node is greater than or equal to 30. SuRe shows the highest accuracies: 10.8% higher MAP, and 5.7% higher Precision@20 compared to the best existing method.

**Fig 7:**
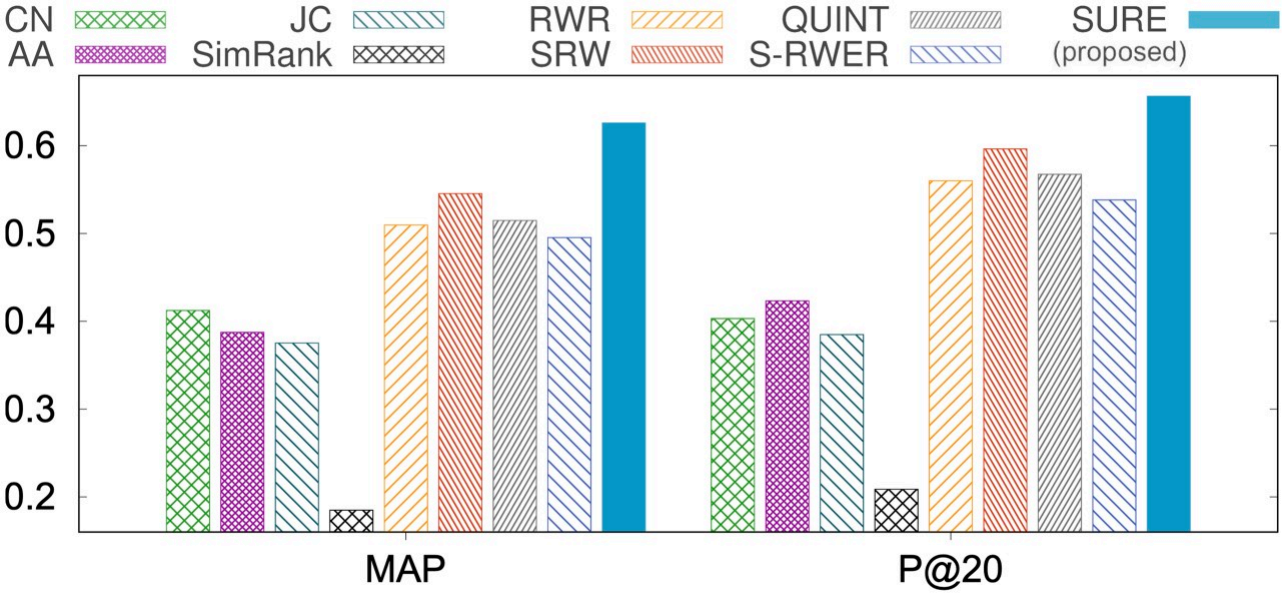
Link prediction performance on the HepTh dataset, where the degree of each query node is greater than or equal to 30. SuRe shows the highest accuracies: 14.7% higher MAP, and 10.1% higher Precision@20 compared to the best existing method.

We divide nodes into two groups; the first group consists of query nodes of non-low degrees whose degrees are greater than or equal to 30, and the second group consists of 176 query nodes of low degrees whose degrees are less than 30. Figs 6, 7 and Table 5 show the link prediction performances of the first group; Tables 6 and 7 show the link prediction performances of the second group.

**Table 5:** AUC result, where the degree of each query node is greater than or equal to 30. We compare SuRe with other baselines. SuRe provides the best link prediction accuracy.

Dataset	SuRe	S-RWER	RWR	SRW	QUINT	SimRank	CN	JC	AA
HepPh	**0.9551**	0.9350	0.9359	0.9441	0.9361	0.4870	0.8913	0.8986	0.9057
HepTh	**0.9603**	0.9306	0.9400	0.9485	0.9420	0.4932	0.8913	0.8986	0.9057

**Table 6:** Link prediction performance on the HepPh dataset, where the degree of each query node is less than 30. Bold and italic fonts indicate the best and the second best methods, respectively.

Evaluation	SuRe	S-RWER	RWR	SRW	QUINT	SimRank	CN	JC	AA
MAP	*0.7109*	0.6845	0.7080	0.7101	0.7045	0.0550	0.6886	0.2178	**0.7329**
AUC	0.9604	0.9589	*0.9609*	**0.9614**	0.9572	0.4870	0.9341	0.8197	0.9560
Pre@20	0.1286	0.1303	*0.1321*	**0.1342**	0.1268	0.0136	0.1198	0.0633	0.1284

**Table 7:** Link prediction performance on the HepTh dataset, where the degree of each query node is less than 30. Bold and italic fonts indicate the best and the second best methods, respectively.

Evaluation	SuRe	S-RWER	RWR	SRW	QUINT	SimRank	CN	JC	AA
MAP	**0.9428**	0.9270	0.9421	*0.9424*	0.9342	0.0577	0.8959	0.5256	0.9188
AUC	**0.9925**	0.9813	**0.9925**	0.9920	0.9859	0.4932	0.9651	0.8226	0.9843
Pre@20	**0.1980**	0.1956	0.1967	*0.1974*	0.1945	0.0139	0.1850	0.1271	0.1885

#### Result on query nodes of non-low degree

Figs 6, 7 and Table 5 show the link prediction performances in terms of MAP, Precision@20, and AUC. Here, we select nodes whose degrees are greater than 30 as query nodes. As shown in the results, our method SuRe outperforms other competitors including SRW and QUINT which are the state-of-the-art methods for link prediction. In the HepTh dataset, compared to the best competitor SRW, SuRe achieves 14.7% improvement in terms of MAP, and 10.1% improvement in terms of Precision@20 (Fig 7). For the AUC results, SuRe gives the best performance as shown in Table 5. AUC is high because of class imbalance (# of positive class ≪ # of negative class) Note that SuRe provides the best prediction over all datasets. The results state that assigning a distinct restart probability to each node and learning the restart probabilities (RWER and SuRe) have a significant effect on link prediction compared to using a fixed restart probability for all nodes (RWR). Furthermore, the result indicates that learning restart probabilities (SuRe) provides better link prediction accuracy than existing supervised learning methods that focus on learning edge weights (SRW) or network topology (QUINT).

We perform a Welch’s *t*-test between the MAP results of SuRe and the best baseline SRW on HepPh. The *p*-value is 0.0038, which indicates the improvement over the baseline is significant.

#### Result on query nodes of low degree

As shown in Tables 6 and 7, SuRe, SRW, and RWR show the best and comparable performances in general for low degree nodes. In the HepTh dataset, SuRe outperforms all other methods in all aspects. In the HepPh dataset, however, other methods including SRW outperform SuRe. We conjecture that this comes from small training instances for low degree nodes, since they have a small number of neighbors.

#### Discussion

We discuss the above experimental results in terms of the number of model parameters. As shown in Table 8, SRW has not enough parameters (i.e., number of user-defined *features* in [5] < number of nodes *n*); thus, feature selection is important for the performance of applications in SRW. On the other hand, QUINT has too many parameters; thus, it is infeasible to learn *O*(*n*^2^) parameters in large-scale graphs. We conjecture that this huge number of parameters is the main reason of the poor performance of QUINT. Compared to these methods, SuRe has a moderate number of parameters, implying that 1) the expressiveness of SuRe is better than that of SRW, and 2) SuRe is not likely to overfit. This point explains why SuRe provides better performance of the ranking and link prediction tasks than SRW and QUINT do as shown in Sections 4.2 and 4.3.

**Table 8:** Number of parameters for each supervised method.

Dataset	SRW [5]	SuRe (proposed)	QUINT [12]
#*parameters*	*O*(#*features*)	*O*(*n*)	*O*(*n*^2^)

### 4.4 Effects of parameters

**Fig 8:**
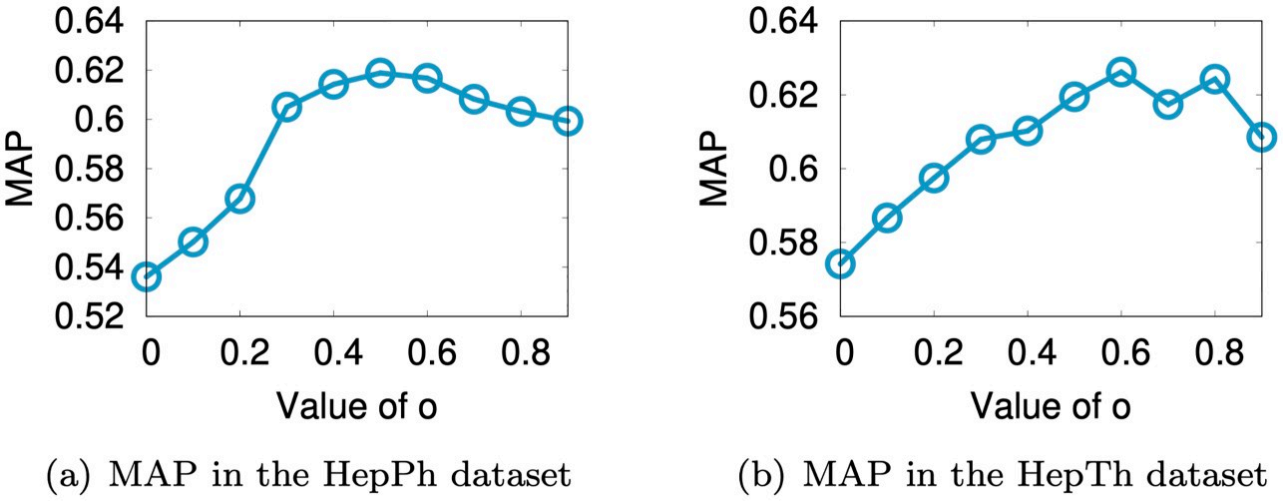
Sensitivity of parameter o of our method SuRe in the HepPh and HepTh datasets. We report the link prediction accuracy using MAP measure, changing the values of the elements in the origin vector **o**, where all the elements of **o** are set to a same value. Note that the performance of SuRe is improved by introducing the origin parameter **o**, compared to not using **o** which corresponds to setting **o** = 0.

We investigate the parameter sensitivity of SuRe w.r.t. the value of the origin vector **o** and λ. The origin vector **o** serves as a model regularizer which helps avoid overfitting and improves accuracy, as described in Section 3.4. We evaluate MAP of the link prediction task, and report the results in Fig 8. Note that the performance of SuRe is improved by introducing the origin parameter **o**, compared to the case without **o**, which corresponds to the leftmost points in both plots of Fig 8.

**Fig 9:**
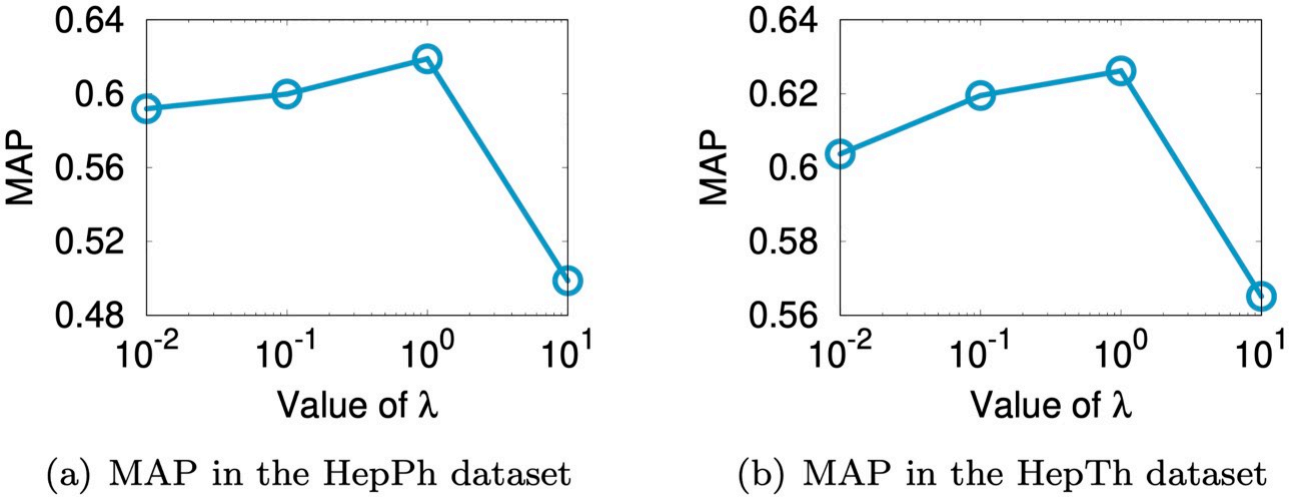
Sensitivity of parameter λ of our method SuRe in the HepPh and HepTh datasets. We report the MAP scores in the link prediction task, varying the value of λ. Note that SuRe avoids overfitting problem and shows improvement by introducing the regularization parameter λ. When λ is 10^0^ = 1, SuRe exhibits the best link prediction performance in both datasets.

λ is a regularization parameter that controls the importance of the regularization term. We evaluate MAP of the link prediction task in the HepPh and HepTh datasets varying the value of λ. As shown in Fig 9, when λ is 1, the MAP score of SuRe is the highest. By introducing the regularization parameter λ, SuRe avoids the overfitting problem and improves accuracy.

### 4.5 Effect of number of labeled nodes

**Fig 10:**
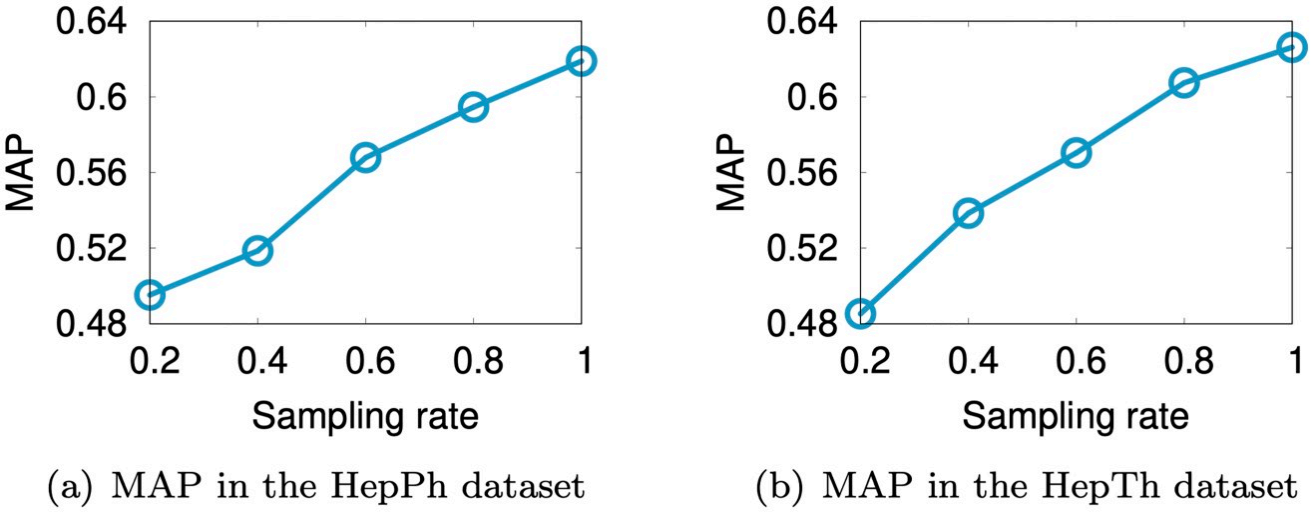
Link prediction performance of SuRe varying the number of labeled nodes in the HepPh and HepTh datasets. We sample labeled nodes with a sampling rate. Note that a higher sampling rate leads to more training examples which in turn improve the performance.

We evaluate the link prediction performance of SuRe by varying the number of labeled nodes. For a given seed node, we sample labeled nodes with a sampling rate. E.g., the average number of the labeled nodes in the Hepth dataset is 69.58; when the sampling rate is 0.2, then the average number of labeled nodes becomes 13.91. As shown in Fig 10, when a seed node has many labeled nodes, SuRe shows better performance since it learns a better model with rich data.

### 4.6 Scalability

**Fig 11:**
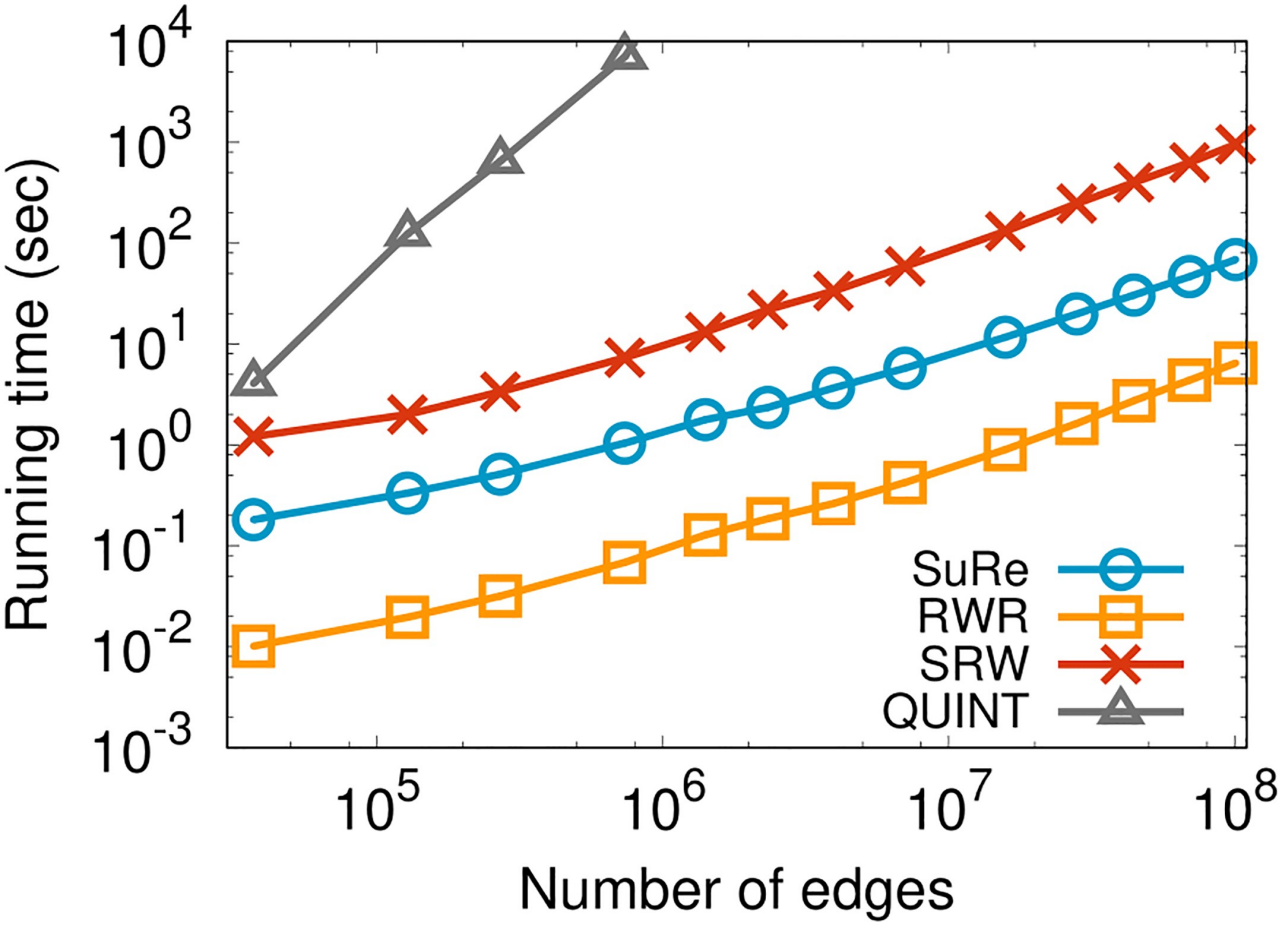
Scalability of SuRe compared to baselines in the Wikipedia dataset. SuRe has the smallest slope 0.76 of the fitted line, while the slopes for RWR, SRW, and QUINT are 0.83, 0.88, and 2.57, respectively. Note that SuRe is the fastest among the supervised methods SRW and QUINT. Although SuRe shows slower running time compared to RWR which is an unsupervised method, SuRe provides higher accuracy than RWR in most cases in both ranking and link prediction tasks as shown in Figs 7, 4 and 6.

We examine the scalability of our proposed method SuRe compared to other baselines. We perform SuRe to find the optimal restart probabilities and RWER to compute rankings with various sizes of the Wikipedia dataset to investigate the scalability of SuRe. For the dataset, we extract the principal sub-matrices, which are the upper left part of the adjacency matrix, of different lengths to get graphs of different number of edges. Fig 11 shows that SuRe scales near-linearly with the number of edges; this result is consistent with Theorem 3. The slope of the fitted line for SuRe is 0.76, the smallest number: those for RWR, SRW, and QUINT are 0.83, 0.88, and 2.57, respectively.

The number of iterations of SuRe is determined by tolerance or the learning rate we set. If we set small tolerance, then the number of iterations will be large. Each SuRe computation for each query node take 39.4 iterations on average in the HepTh dataset. Note that SuRe is the fastest among the supervised methods. Although RWR, which is an unsupervised method, is faster than SuRe, RWR shows low accuracy as shown in Figs 7, 4 and 6.

## 5 Related works

The related works fall into two main categories: 1) relevance measures in graphs, 2) label prediction and classification, and 3) ranking and link prediction based on relevance measures.

### Relevance measures in graphs

There are various relevance measures in graphs based on link analysis and random walk, e.g., PageRank [27], HITS [4], SimRank [26], Random Walk Graph Kernel [28], and RWR (or Personalized PageRank) [6]. Among these measures, RWR has received much attention from the data mining community since it provides a personalized ranking w.r.t. a node, and it has been applied to many graph mining applications such as community detection [13], link prediction [5, 12], ranking [9], and graph matching [29]. Also, fast and scalable methods [10, 11, 9] for computing RWR in large graphs have been proposed to boost the performance of those applications in terms of time.

### Label prediction and classification

Considering the problem setting, our work is related to label propagation. Hwang and Kuang [30] proposed the MINProp algorithm, a heterogeneous label propagation algorithm to discover disease genes. This algorithm is based on [31], whose algorithm spreads label information. These problems assume the graphs are undirected, but our graphs in the experiments are directed. Vanunu et al. [25] proposed a prioritization algorithm, which prioritize a set of genes in protein-protein interaction networks. Sousa et al. [32] studied graph-based semi-supervised learning algorithms and graph construction methods on labeled graphs. Ji et al. [33] proposed a graph-based regularization framework to model the link structure in information networks. They treated different types of objects and links separately because of different semantic meanings. They also proposed a ranking-based classification algorithm [34], which integrates ranking and classification. Sen et al. [35] provided a brief description of some of most widely used classification algorithms for classifying networked data.

### Ranking and link prediction

Jung et al. [36] extended the concept of RWR to design a personalized ranking model in signed networks. Our proposed algorithm is different from SRWR in that SRWR is for edge-labeled graphs. Regarding the edge-labeled graphs, there are some works on knowledge graphs [37, 38, 39]. Lao et al. [37] extended the Path Ranking algorithm adjusting the weights associated with random walks that follow different paths through the graph. Wang et al. [38] developed a fast and easily-parallelized weight-learning algorithm for ProPPR, based on local partitioning methods. Wei et al. [39] introduced a goal-directed random walk algorithm which directs random walks by a specific inference target. Wang et al. [40] proposed an image annotation technique that generates candidate annotations and re-ranks them using RWR. Pan et al. [41] exploited RWR to discover correlations across multimedia data. Sun et al. [15] used RWR ranking results for detecting anomalies in bipartite networks. Gleich et al. [42] have empirically shown that random walk based models such as RWR is competitive with other cut based approaches for detecting local communities in graphs. Liben-Nowell et al. [23] extensively studied the link prediction problem in social networks based on relevance measures such as PageRank, RWR, and Adamic-Adar [1]. Grover and Leskovec [43] proposed a representation learning algorithm based on random walks. This work can be utilized in link prediction. Many researchers have proposed supervised learning methods for link prediction. Backstrom et al. [5] proposed Supervised Random Walk (SRW), a supervised learning method for link prediction based on RWR. SRW learns parameters for adjusting edge weights. Li et al. [12] developed QUINT, a learning method for finding a query-specific optimal network. QUINT modifies the network topology including edge weights.

In many real-world scenarios, however, modifying the graph structure would not be allowed. On the contrary, our SuRe method controls the behavior of the random surfer without modifying the graph structure, and provides better prediction accuracy than other competitors as shown in Section 4.

## 6 Conclusion

We propose Random Walk with Extended Restart (RWER), a novel relevance measure using distinct restart probabilities for each node. We also propose SuRe, a data-driven algorithm for learning restart probabilities of RWER. Experiments show that our method brings the best performance for ranking and link prediction tasks, outperforming the traditional RWR and recent supervised learning methods. Future works include extending the proposed method for heterogeneous and edge labeled graphs.
