# Correction: Supervised and extended restart in random walks for ranking and link prediction in networks

**DOI:** 10.1371/journal.pone.0216572

**Published:** 2019-05-01

**Authors:** The PLOS ONE Staff

There are errors in the Funding statement. The publisher apologizes for the errors. The correct Funding statement is as follows: This work was supported by Institute of Information & Communications Technology Planning & Evaluation (IITP) grant funded by the Korea government (MSIT) [2013-0-00179, Development of Core Technology for Context-aware Deep-Symbolic Hybrid Learning and Construction of Language Resources]. The Institute of Engineering Research at Seoul National University provided research facilities for this work. The ICT at Seoul National University provides research facilities for this study. The funders had no role in study design, data collection and analysis, decision to publish, or preparation of the manuscript.
